# Exposure to Phenols, Phthalates, and Parabens and Development of Metabolic Syndrome Among Mexican Women in Midlife

**DOI:** 10.3389/fpubh.2021.620769

**Published:** 2021-02-26

**Authors:** Astrid N. Zamora, Erica C. Jansen, Marcela Tamayo-Ortiz, Jaclyn M. Goodrich, Brisa N. Sánchez, Deborah J. Watkins, Juan Alfredo Tamayo-Orozco, Martha M. Téllez-Rojo, Adriana Mercado-García, Ana Baylin, John D. Meeker, Karen E. Peterson

**Affiliations:** ^1^Department of Nutritional Sciences, University of Michigan School of Public Health, Ann Arbor, MI, United States; ^2^Occupational Health Research Unit, Mexican Social Security Institute, Mexico City, Mexico; ^3^Department of Environmental Health Sciences, University of Michigan School of Public Health, Ann Arbor, MI, United States; ^4^Department of Epidemiology and Biostatistics, Drexel University Dornsife School of Public Health, Philadelphia, PA, United States; ^5^Accessalud, Mexico City, Mexico; ^6^National Institute of Public Health, Mexico City, Mexico; ^7^Department of Epidemiology, University of Michigan School of Public Health, Ann Arbor, MI, United States

**Keywords:** endocrine disrupting chemicals, midlife women, metabolic syndrome, phenol, paraben, phthalate, environmental exposures

## Abstract

**Background:** Evidence suggests exposure to endocrine-disrupting chemicals (EDCs) can influence Metabolic Syndrome (MetS) risk in adults, but it is unclear if EDCs impact women during midlife. We examined if EDCs measured in adult women were predictive of MetS and its components 9 years later.

**Methods:** We measured urinary phthalate metabolites, phenols, and parabens collected in 2008 among 73 females from the ELEMENT study. MetS and its components (Abdominal Obesity, Hypertriglyceridemia, Cholesterolemia, Hypertension, and Hyperglycemia) were assessed in 2017. We regressed log-transformed EDC concentrations on MetS and MetS components using logistic regression, adjusting for age and physical activity.

**Results:** At follow-up, the mean (SD) age was 46.6 (6.3) years; the prevalence of MetS was 34.3%. Sum of dibutyl phthalate metabolites (ΣDBP), monobenzyl phthalate (MBzP), and monoethyl phthalate (MEP) were associated with an increased odds of hypertriglyceridemia. 2,5-dichlorophenol (2,5 DCP) and 2,4-dichlorophenol (2,4 DCP) were associated with increased odds of hypertriglyceridemia. The odds of hypertension were 4.18 (95% CI: 0.98, 17.7, *p* < 0.10) and 3.77 (95% CI: 0.76, 18.62, *p* < 0.10) times higher for every IQR increase in MCOP and propyl paraben, respectively. The odds of hyperglycemia were 0.46 (95% CI: 0.18, 1.17 *p* < 0.10) times lower for every IQR increase in the sum of di-2-ethylhexyl phthalate metabolites (ΣDEHP), and the odds of abdominal obesity were 0.70 (95% CI: 0.40, 1.21, *p* < 0.10) lower for every IQR increase in the concentration of triclosan.

**Conclusion:** We found EDCs measured in 2008 were marginally predictive of hypertriglyceridemia and hypertension 9 years later. Results suggest that lower exposure to certain toxicants was related to lower markers of metabolic risk among midlife women.

## Introduction

Metabolic syndrome (MetS) is a clinical and public health issue that is associated with an increased risk for several adverse health effects, including a predisposition to Type 2 Diabetes (T2D) and Cardiovascular Disease (CVD) (1). For women, the midlife period is often when MetS and its related sequelae manifest (2, 3). Midlife for women encompasses the menopausal transition, a sensitive period for women that is characterized by fluctuating hormone levels and rapid physiological and psychological symptoms rooted in hormonal alterations (4). During the menopausal transition, women may increase their body weight by 20–25% (5). Other studies have found a similar association between abdominal obesity during midlife (6, 7). Furthermore, during this rapid period marked by hormonal imbalance, abdominal obesity favors insulin resistance (8), and the risk of hypertension and other chronic conditions increases (5). Due in part to the changing hormonal milieu present during this period, midlife may be especially sensitive to toxicant effects, thus there is an increasing interest in how long-term exposure to endocrine-disrupting chemicals (EDCs) may affect risk of MetS at midlife (9).

Phthalate metabolites, phenols, and parabens are three chemical classes that may be related to MetS. One plausible underlying mechanism that may explain the relationship between EDC exposure and MetS is via EDCs mimicking natural steroid hormones, thus enabling EDCs to interact with steroid hormone receptors as analogs or antagonists (10). In particular, there is evidence of phthalates modulating hormones and inflammatory pathways that are known to be involved in MetS (11). Another plausible mechanism is through oxidative stress (12). Recent cross-sectional studies have provided evidence of positive associations between MetS and Bisphenol A (BPA) among women over the age of 40 in Lebanon (13) and MetS and Monobenzyl phthalate (MBzP) among premenopausal women in the US (11). Cross-sectional studies in American, Asian, and Middle Eastern women demonstrated positive associations between exposure to phenols (14–18) and phthalates (11, 19–21) with components of MetS (Abdominal Obesity, Hypertriglyceridemia, Cholesteremia, Hypertension, and Hyperglycemia). However, a recent cross-sectional study among a nationally representative sample of Canadian women found conflicting associations between several parabens and cardio-metabolic outcomes. These findings included inverse associations between ethyl paraben and MetS; methyl paraben with obesity; and positive associations between methyl, propyl, ethyl parabens, and higher-density concentrations lipoprotein (HDL) cholesterol (22). These inconsistent findings across numerous cross-sectional studies call for prospective research studies.

Relationships between EDCs and MetS among midlife women are understudied in Latin American populations. Most Latin American research focuses on the relationship between MetS prevalence and sociodemographic factors (23, 24) or the relationship between toxicants and other metabolic parameters in children (25). Thus, this pilot study among Mexican women aimed to analyze if exposure to phenols, phthalates, and parabens were prospectively associated with MetS and its components measured ~9 years later when women were in midlife and of peri-/menopausal age.

## Materials and Methods

The study population included a sample of Mexican women initially re-recruited in 2008 from three-consecutive cohorts of the Early Life Exposure in Mexico to ENvironmental Toxicants (ELEMENT) study (26). Women were originally recruited beginning in 1994, either during pregnancy or at delivery depending on the cohort, from family clinics of the Mexican Institute of Social Security, serving a homogenous, low-income to middle-income population in Mexico City. At the 2008 follow-up visit, we collected biological samples, including a spot urine sample stored for future analyses. Between May and October 2017, we re-recruited 101 women to assess lifestyle factors and MetS components. The sample of 101 women included participants from each of the three consecutive cohorts (Cohort 1: *n* = 29; Cohort 2: *n* = 22; Cohort 3: *n* = 50). In 2019, we measured the concentration of phthalate metabolites, phenols, and parabens in samples collected in 2008. Further, we evaluated the association between concentrations of the toxicants and MetS and its components (Abdominal Obesity, Hypertriglyceridemia, Cholesterolemia, Hypertension, and Hyperglycemia) 9 years later among 73 women (the final sample size was <101 obtained from random sampling due to budget constraints). Subject recruitment and procedures for participants in the ELEMENT study have been described elsewhere (26). The Mexico National Institute of Public Health (INSP) and the University of Michigan Internal Review Boards approved all research protocols and procedures, and all participants provided informed consent.

### Exposures

Using urine samples stored at −80°C, concentrations for 24 EDCs were measured using isotope dilution–liquid chromatography-tandem mass spectrometry (ID–LC-MS/MS) at NSF International (Ann Arbor, MI, USA). This method was developed based on the Centers for Disease Control and Prevention (CDC) methods for measuring phenols, phthalates, and parabens from urinary samples (27, 28). It was evaluated against acceptance criteria established within CDC methods. EDCs included 16 phthalate metabolites: metabolites of DBP (MBP, MiBP), metabolites of DEHP (MEHP, MEHHP, MEOHP, MECPP), MECPTP, MEHHTP, MBzP, MCNP, MCOP, MCPP, MEP, MNP, cxMINCH, and OH-MINCH; 4 parabens (Butyl Paraben, Ethyl Paraben, Methyl Paraben, and Propyl Paraben); and 8 phenols (2,4 DCP, 2,5 DCP, Triclocarban, Triclosan, BP3, BPA, BPF, BPS. We calculated a DBP metabolite summary measure (ΣDBP) for each sample by dividing individual MBP and MiBP concentrations by their molecular mass and summing them. The same was done to create the DEHP metabolite summary measure (ΣDEHP) for each sample, dividing individual MEHP, MEHHP, MEOHP, and MECPP concentrations by their molecular mass and summing them. Values below the limit of detection (LOD) were replaced with the metabolite-specific LOD/√2.

### Outcomes

Outcomes were assessed in 2017 at a clinic visit. The rationale for measuring the outcome in 2017 was primarily due to budgetary reasons and because the present study was considered a pilot study to generate evidence for future grants. At this visit, trained research staff collected weight, height, and waist circumference using standard methods (29). Body-mass-index (BMI) was calculated as kg/m^2^, and waist circumference was measured to the nearest 0.1 cm. Fasting venous blood samples were collected, and serum separated to assess glucose, triglycerides, total cholesterol, and high-density lipoprotein (HDL) cholesterol. These markers were measured enzymatically with correction for endogenous glycerol at INPer (Institute of Perinatology, Mexico). Blood pressure (diastolic and systolic) was measured by trained personnel using a standardized protocol with a Spacelabs Healthcare automated oscillometric device. To assess MetS and individual MetS components, we used the National Cholesterol Education Program (NCEP) Adult Treatment Panel-III (ATP-III) definition to classify MetS, i.e., ≥3 of 5 components: Abdominal Obesity (waist circumference >88 cm); Hypertriglyceridemia (serum triglycerides ≥150 mg/dL); Cholesterolemia (serum HDL cholesterol <50 mg/dL); Hypertension—systolic blood pressure of ≥130 mmHg and/or diastolic blood pressure ≥85 mmHg), and Hyperglycemia (fasting serum glucose ≥100 mg/dL) (30).

### Covariates

We selected covariates based on a *priori* knowledge. Information for potential confounders was collected through a demographic questionnaire administered at baseline. Participant's age (years) and maternal education (years) were operationalized as continuous variables. Physical activity was measured with the International Physical Activity Questionnaires (IPAQ) (31). We used self-reported participation in moderate physical activity within the past 7 days: No for 0 days and Yes for 1–7 days. A dichotomous variable for menopausal status was created based on self-report. Pre-menopause was defined as having a period or menstruation within the past 12 months, while peri-/post menopause was classified as an absence of a period or menstruation ≥12 months (32). Finally, lifetime smoking status was classified as a categorical variable, whether women self-reported as a current smoker, previous smoker, or never smoker in their lifetime.

### Statistical Analysis

We calculated summary statistics (mean and standard deviation or proportion) of sociodemographic, behavioral, and metabolic characteristics, including age, BMI, waist circumference, diastolic and systolic blood pressure, triglycerides, HDL cholesterol, fasting blood glucose, maternal education, participation in moderate physical activity, menopausal status, and lifetime smoking status stratified by MetS status. To test for differences between MetS status, we used a student's *t*-test for continuous variables that were normally distributed, Wilcoxon rank test for continuous variables that were not normally distributed, and a chi-squared test for categorical variables. To assess exposure to EDCs in 2008, we calculated arithmetic means, standard deviations, and percentiles to describe the distribution of urinary biomarker concentrations. All EDC biomarkers and covariates were then evaluated for non-normality and were log-transformed before analysis to reduce the influence of outliers. Phenol, phthalate, and paraben analytes were creatinine-adjusted to correct urine diluteness variations (33). We completed regression diagnostics to evaluate model assumption and determine presence of outliers and influential points. For analytes detected in more than 75 percent of samples, we generated logistic regression models in which MetS and individual MetS components served as the outcome of interest, while the different phenol, phthalate, and paraben analytes were considered as continuous predictor variables. Because EDC concentrations were non-normally distributed, we present estimates per interquartile range (IQR) concentrations in all regression models. Based on the bivariate analysis results, all models were adjusted for participant's age and participation in moderate physical activity. In sensitivity analyses, menopausal status was included in regression models. Findings were considered statistically significant at *p* < 0.05 and marginally significant at *p* < 0.10. All statistical analyses were conducted using SAS 9.4 software (Cary, NC, USA).

## Results

Among the 73 women included in the present study, data from 2017 demonstrated that the mean (SD) age was 46.6 (6.3) years, with a mean (SD) BMI of 29.4 (4.9) kg/m^2^. Further, the mean (SD) for each MetS component was: waist circumference 92.1 (11.0) cm, triglycerides 165.9 (87.9) mg/dL, HDL cholesterol 43.6 (7.7) mg/dL, systolic blood pressure 117.8 (17.7) mmHg, diastolic blood pressure 70.2 (11.8) mmHg, and fasting blood glucose 88.4 (44.1) mg/dL. Thirty-four percent (34.3%; *n* = 25) of the women had MetS. Some characteristics slightly differed between women with MetS and women without MetS, including age and self-reported participation in moderate physical activity. Specifically, women in the MetS group were ~3 years older than the non-MetS group. Further, 12.5% of women in the MetS group reported participating in moderate physical activity, compared to 31.9% of women in the non-MetS group. Maternal education and lifetime smoking status were not significantly different between the MetS group and non-MetS group. Although 34.2% of the entire sample were in the peri-/menopause stage, menopause status was not significantly different between the MetS and non-MetS group (Table 1).

**Table 1:** Population characteristics by Metabolic Syndrome status (*n* = 73).

	**Non-MetS (*n* = 48)**	**MetS (*n* = 25)**	***P*-value**
**MEAN (SD)**			
Age (years)	45.7 (6.4)	48.3 (6.0)	0.0896^*^
BMI (kg/m^2^)	28.0 (4.9)	32.0 (3.8)	0.0004^**^
Waist Cir. (cm)	89.5 (11.1)	97.2 (1.8)	0.0035^**^
Diastolic BP (mmHg)	67.4 (10.0)	75.4 (12.2)	0.0025^**^
Systolic BP (mmHg)	113.8 (13.4)	125.2 (14.0)	< 0.0001^***^
Triglycerides	132.4 (60.3)	230.2 (97.4)	< 0.0001^***^
HDL cholesterol	45.4 (8.2)	40.3 (5.4)	0.0025^**^
Fasting glucose	86.2 (50.3)	92.7 (21.2)	0.0660^*^
Maternal education (years)	9.9 (3.6)	9.4 (2.7)	0.5904
**Menopausal status**			
*Pre-*	68.2%	60.0%	0.4928
*Peri-/post*	31.8%	40.0%	
**Moderate Physical Activity**			0.0753^*^
*No*	68.1%	87.5%	
*Yes*	31.9%	12.5%	
**Lifetime Smoking**			0.7880
*Never Smoker*	53.3%	54.6%	
*Former Smoker*	20.0%	13.6%	
*Current Smoker*	26.7%	31.8%	

*** *p < 0.0001 from Wilcoxon rank test*,

** *p < 0.05 (2-tailed) from Wilcoxon rank test or Student's t-test*,

* *p < 0.10 from Wilcoxon rank test, Chi-square test or Student's t-test*.

*ABR: MetS, Metabolic Syndrome; SD, Standard Deviation; BMI, Body-Mass-Index; Cir, Circumference; BP, Blood Pressure; HDL, High Density Lipoprotein*.

### Distribution of EDCs

Except for 5 phthalate metabolites (MEHHTP, MCNP, MNP, cxMINCH, OH-MINCH), 3 phenol analytes (Triclocarban, BPF, BPS), and 2 paraben analytes (Butyl Paraben and Ethyl Paraben), the remaining EDCs were detected in ≥75 percent of the urine samples, representing widespread exposure among participants. Phthalate metabolites, MECPP, MEHHP, MEOHP, MBzP, MBP, MEP, and MiBP, were detected in 100 percent of the samples. Further, 2,5 DCP, 2,4 DCP, Triclosan, BP3, and BPA were detected in 100, 98.6, 75.3, 98.6, and 83.6 percent of the samples, respectively. Among the samples analyzed, we detected Methyl Paraben and Propyl Paraben in 100 percent of the samples (Table 2). Subsequent regression analyses were only performed among analytes detected in at least 75 percent of the urine samples.

**Table 2:** Summary statistics for creatinine-adjusted urinary phthalate metabolites, phenols and parabens analyzed (2019) from spot urine collected in 2008.

	**LOD**	**% < LOD**	**Arithmetic mean ± SD (ng/mL)**	**Minimum**	**25th**	**75th**	**Maximum**
ΣDBP^a^	–	–	126.9 ± 211.1	6.6	36.4	142.0	1731.2
ΣDEHP^b^	–	–	139.7 ± 285.7	4.5	45.2	138.6	2368.0
MECPP	0.2	0	65.9 ± 122.0	1.7	24.4	71.2	1035.2
MEHHP	0.1	0	49.6 ± 110.6	1.6	13.4	47.2	881.4
MEHP	1	6.9	7.5 ± 14.9	0.7	1.7	6.8	116.5
MEOHP	0.1	0	27.9 ± 63.1	0.8	7.7	25.4	520.3
MECPTP	0.2	2.7	3.6 ± 5.7	0.1	0.9	4.3	44.9
MEHHTP	0.2	32.9	0.5 ± 0.8	0.1	0.1	0.6	5.2
MBzP	0.2	0	8.6 ± 10.7	0.3	2.5	9.9	47.6
MBP	0.5	0	115.4 ± 205.0	5.0	31.0	128.4	1693.0
MCNP	0.2	71.2	0.2 ± 0.1	0.1	0.1	0.2	0.6
MCOP	0.2	8.2	1.0 ± 1.1	0.1	0.4	1.2	5.6
MCPP	0.2	2.7	1.8 ± 1.9	0.1	0.6	2.1	10.9
MEP	1.0	0	369.1 ± 480.4	11.8	56.7	449.4	2056.6
MNP	0.5	100	0.4 ± 0.0	0.4	0.4	0.4	0.4
MiBP	0.1	0	11.4 ± 11.7	0.5	4.2	13.6	71.1
cxMINCH	0.2	87.7	0.3 ± 0.9	0.1	0.1	0.1	8.0
OH-MINCH	0.2	75.2	0.3 ± 1.3	0.1	0.1	0.1	11.1
2,4 DCP	0.2	1.4	16.9 ± 42.2	0.1	0.8	5.3	224.4
2,5 DCP	0.2	0	206.5 ± 568.5	0.9	6.2	46.3	3092.0
Triclocarban	2	90.4	1.6 ± 0.8	1.4	1.4	1.4	7.6
Triclosan	2	24.7	93.3 ± 236.5	1.4	2.1	19.7	1373.6
BP3	0.4	1.4	278.0 ± 1019.2	0.3	3.0	103.0	6105.8
BPA	0.4	16.4	1.2 ± 0.9	0.3	0.6	1.5	4.4
BPF	0.4	95.9	0.4 ± 0.8	0.3	0.3	0.3	6.8
BPS	0.4	87.7	0.5 ± 1.3	0.3	0.3	0.3	11.5
Butyl Paraben	0.2	60.3	2.6 ± 9.9	0.1	0.1	0.6	77.7
Methyl Paraben	1	0	55.3 ± 169.3	0.7	0.7	20.2	1262.7
Ethyl Paraben	1	34.3	370.8 ± 442.3	1.7	62.6	459.5	1802.7
Propyl Paraben	0.2	0	127.8 ± 202.2	0.2	10.4	133.6	1212.1

*ABR: LOD, limit of detection; DBP, dibutyl phthalate metabolites; DEHP, di-2-ethylhexyl phthalate metabolites; MECPP, mono-2-ethyl-5-carboxypentyl phthalate; MEHHP, mono-2-ethyl-5-hydroxyhexyl phthalate; MEHP, mono-2-ethylhexyl phthalate; MEOH, mono-2-ethyl-5-oxohexyl phthalate; MECPTP, Mono-2-ethyl-5-carboxypentyl Terephthalate-d4; MEHHTP, mono-2-ethyl-5-hydroxyhexyl terephthalate; MBzP, monobenzyl phthalate; MBP, Mono-n-butyl phthalate; MCNP, Monocarboxy-isononly phthalate; MCOP, Monocarboxyoctyl phthalate; MCPP, mono-3-carboxypropyl phthalate; MEP, monoethyl phthalate; MNP, Mono-isononyl phthalate; MiBP, mono-isobutyl phthalate; cxMINCH, cyclohexane-1,2-dicarboxylic mono carboxyisooctyl ester; OH-MINCH, cyclohexane-1,2-dicarboxylic mono hydroxyisononyl ester; 2,4 DCP, 2,4-Dichlorophenol; 2,5 DCP:2,5-Dichlorophenol; BP3, Benzophenone-3; BPA, Bisphenol A; BPS, Bisphenol S; BPF, Bisphenol F*.

a *ΣDBP: molar sum of MBP, MiBP*;

b *ΣDEHP: molar sum of MEHP, MEHHP, MEOHP, and MECPP*.

### Phthalate Findings

There were no associations between phthalates and overall MetS. However, several phthalate metabolites were positively associated with hypertriglyceridemia. Adjusted logistic regression demonstrated that for every one IQR increase in dibutyl phthalate metabolites (ΣDPB), monobenzyl phthalate (MBzP), and monoethyl phthalate (MEP), the odds of hypertriglyceridemia were 2.04 (95% CI: 1.03, 4.03, *p* < 0.05), 1.72 (95% CI: 0.89, 3.29, *p* < 0.10) and 2.17 (95% CI: 1.01, 4.66, *p* < 0.05) times higher. We observed a similar pattern among other phthalate metabolites, suggesting a positive relationship with hypertriglyceridemia, though results were not statistically significant. Additionally, for every one IQR increase in the concentration of monocarboxyoctyl phthalate (MCOP), the odds of hypertension were 4.18 (95% CI: 0.98, 17.81, *p* < 0.10) times higher. In accordance, we observed a similar pattern among all measured phthalates, demonstrating a positive association with hypertension, though not statistically significant (Table 3). Further, adjusted logistic regression analysis demonstrated that for every one interquartile range (IQR) increase in the concentration of di-2-ethylhexyl phthalate metabolites (ΣDEHP), the odds of hyperglycemia were 0.46 (95% CI: 0.18, 1.17, *p* < 0.10) times lower. In accordance, we observed a similar pattern among all other phthalates, demonstrating an inverse association with hyperglycemia, though not statistically significant (Table 3).

**Table 3:** Adjusted logistic regression analysis of EDCs to individual Metabolic Syndrome components and Metabolic Syndrome.

**Parent comps. and metabolites**	**Abdominal obesity**	**Hypertriglyceridemia**	**Cholesteremia**	**Hypertension**	**Hyperglycemia**	**MetS**
**OR (95% CI)**^***a***^, ^***b***^						
ΣDBP^c^	1.55 (0.81, 2.97)	2.04 (1.03, 4.03)^**^	1.59 (0.72, 3.51)	1.42 (0.43, 4.66)	0.80 (0.28, 2.27)	1.78 (0.86, 3.65)
ΣDEHP^d^	0.88 (0.51, 1.50)	1.05 (0.62, 1.80)	1.07 (0.55, 2.09)	1.84 (0.63, 5.35)	0.46 (0.18, 1.17)^*^	1.16 (0.65, 2.08)
MECPTP	1.23 (0.62, 2.43)	1.34 (0.68, 2.67)	0.98 (0.42, 2.31)	3.50 (0.71, 17.14)	0.62 (0.20, 1.92)	1.03 (0.50, 2.11)
MBzP	1.32 (0.71, 2.48)	1.72 (0.89, 3.29)^*^	1.51 (0.70, 3.26)	1.21 (0.41, 3.57)	0.63 (0.22, 1.81)	1.60 (0.80, 3.23)
MCOP	1.20 (0.61, 2.37)	1.17 (0.60, 2.30)	1.10 (0.48, 2.51)	4.18 (0.98, 17.71)^*^	0.59 (0.18, 1.89)	1.37 (0.65, 2.86)
MCPP	1.74 (0.85, 3.56)	1.61 (0.79, 3.25)	1.50 (0.62, 3.65)	1.79 (0.46, 7.03)	0.44 (0.14, 1.42)	1.49 (0.70, 3.17)
MEP	0.81 (0.39, 1.67)	2.17 (1.01, 4.66)^**^	1.03 (0.42, 2.52)	1.06 (0.27, 4.22)	0.98 (0.30, 3.16)	1.55 (0.71, 3.39)
2,5 DCP	1.18 (0.68, 2.03)	1.74 (0.98, 3.05)^*^	1.37 (0.66, 2.86)	0.76 (0.25, 2.31)	1.09 (0.47, 2.51)	1.55 (0.87, 2.76)
2,4 DCP	1.20 (0.68, 2.13)	1.78 (0.99, 3.23)^*^	1.54 (0.71, 3.34)	0.63 (0.19, 2.03)	1.07 (0.45, 2.58)	1.54 (0.84, 2.80)
Triclosan	0.70 (0.40, 1.21)^*^	0.88 (0.51, 1.52)	1.40 (0.63, 3.11)	1.20 (0.41, 3.52)	0.49 (0.13, 1.80)	0.85 (0.46, 1.57)
BP3	0.84 (0.40, 1.79)	0.85 (0.41, 1.77)	1.32 (0.50, 3.52)	1.15 (0.30, 4.44)	0.33 (0.06, 1.74)	0.79 (0.36, 1.73)
BPA	1.30 (0.67, 2.54)	1.04 (0.56, 1.91)	1.52 (0.67, 3.46)	0.73 (0.23, 2.35)	0.62 (0.21, 1.85)	1.13 (0.57, 2.22)
Methyl Paraben	0.92 (0.48, 1.76)	1.40 (0.73, 2.69)	1.41 (0.60, 3.28)	2.93 (0.52, 16.45)	0.86 (0.32, 2.30)	1.24 (0.61, 2.52)
Propyl Paraben	1.20 (0.63, 2.32)	1.45 (0.73, 2.87)	1.10 (0.46, 2.64)	3.77 (0.76, 18.62)^*^	0.65 (0.23, 1.86)	1.60 (0.74, 3.45)

** *p <0.05 (2-tailed)*,

* *p < 0.10 (2-tailed)*.

a *Estimates adjusted for age and moderate physical activity level*;

b *Natural-log (ln) transformed*;

c *ΣDBP: molecular sum of MBP, MiBP*;

d *ΣDEHP: molecular sum of MEHP, MEHHP, MEOHP, and MECPP*.

*ABR: EDCs, Endocrine-Disrupting Chemicals; MetS, Metabolic Syndrome; comps, compounds; CI, confidence interval; DBP, dibutyl phthalate metabolites; DEHP, di-2-ethylhexyl phthalate metabolites; MECPTP, Mono-2-ethyl-5-carboxypentyl Terephthalate-d4, MBzP, monobenzyl phthalate; MCOP, Monocarboxyoctyl phthalate; MCPP, mono-3-carboxypropyl phthalate; MEP, monoethyl phthalate; 2,5 DCP, 2,5-Dichlorophenol; 2,4 DCP, 2,4-Dichlorophenol; BP3, Benzophenone-3; BPA, Bisphenol A*.

### Paraben Findings

Overall, adjusted logistic regression analysis did not provide evidence between exposure to parabens and the presence of MetS after 9 years. However, findings revealed that the odds ratio for hypertension was 3.77 (95% CI: 0.76, 18.62, *p* < 0.10) higher for every one IQR increase in propyl paraben, after we adjusted for participant age and moderate physical activity level (Table 3). Methyl paraben demonstrated the same association with hypertension, though findings were not statistically significant.

### Phenol Findings

Adjusted logistic regression analysis demonstrated no statistically significant associations between phenols and MetS. With regards to hypertriglyceridemia, findings revealed that for every one IQR increase in 2,5-dichlorophenol (2,5 DCP) and 2,4-dichlorophenol (2,4 DCP), the odds of hypertriglyceridemia were 1.74 (95% CI: 0.98, 3.05, *p* < 0.10) and 1.78 (95% CI: 0.99, 3.23, *p* < 0.10) higher, respectively. Yet, in analysis examining individual components of MetS, triclosan was associated with a lower odds of abdominal obesity, with 0.70 (95% CI: 0.40, 1.21, *p* < 0.10) lower odds of abdominal obesity for every one unit IQR increase (Table 3). No other phenols were associated with individual components of MetS.

### Sensitivity Analysis

Among the sample, 34.2% (*n* = 25) of women were in the peri-/menopausal stage, compared to 65.8% (*n* = 48) pre-menopausal women. In sensitivity analyses, additional adjustment for menopausal status did not alter regression estimates or affect overall results. Thus, the final adjusted regression models only included participant's age and participation in moderate physical activity.

## Discussion

The present pilot study aimed to explore if concentrations of urinary phthalate metabolites, phenols, and parabens collected in 2008 among Mexican women were associated with MetS and its components 9 years later during midlife age. Our results provide evidence that EDCs may affect individual components of MetS, particularly hypertriglyceridemia and hypertension, in a dose-response manner.

When we analyzed phthalate metabolites with individual components of MetS, we demonstrated a significant positive association between dibutyl phthalate metabolites (ΣDBP), MBzP, and MEP exposure in 2008 with prospective hypertriglyceridemia. However, a longitudinal study among US women of reproductive age did not find an association between MEP and hypertriglyceridemia (34). One reason for this disagreement in findings could be due to differences in study populations, namely that women in the present sample were in the sensitive period of midlife, while the other sample was women of reproductive age. Further, adjusted logistic regression findings provided evidence that exposure to MCOP in 2008 was predictive of hypertension ~9 years later. Our findings align with previous cross-sectional research, which demonstrated a similar association between MCOP and diastolic blood pressure among US children and adolescents (35, 36). Similarly, we found that MEHHP was associated with increased diastolic blood pressure; these findings align with those among a cohort of Chinese children (37). These findings collectively may suggest that associations between toxicants and blood pressure begin earlier in life and are consistent through adulthood. We found an unexpected association whereby ∑DEHP exposure was inversely associated with hyperglycemia. This finding is in line with cross-sectional results among a nationally representative study (NHANES 2001–2008) of American women (38). Albeit, our findings were contrary to a different cross-sectional study conducted among American women (NHANES 2001–2010) (11), which found an increased odds of hyperglycemia with increasing concentration of ∑DEHP, though results were not statistically significant. Similarly, among another NHANES study, ∑DEHP exhibited a strong positive association with fasting blood glucose levels (i.e., hyperglycemia). However, results were obtained for an adult population and not stratified by sex (39).

Our findings did not reveal statistically significant associations between individual parabens and MetS among the cohort. However, we found a positive association between propyl paraben and hypertension, with a magnitude of over 3-fold higher odds. These findings differ from results obtained from a cross-sectional nationally representative sample of US adults (2011–2012) (40). Although not statistically significant, both parabens measured in 2008 were positively associated with hypertension, while findings from the same 2011–2012 NHANES study did not demonstrate a clear pattern (36).

In analyzing the relationship between phenol metabolites and MetS using a prospective study design, phenol metabolites were not significantly associated with MetS. However, 2,5 DCP and 2,4 DCP were associated with increased odds of hypertriglyceridemia among the sample. These findings are contrary to study results among a nationally representative sample of non-diabetic US adults (NHANES 2007–2010), which did not observe associations between DCPs and hypertriglyceridemia (41). Though it is unclear why these differences exist, it is plausible that mean levels of DCP exposure were different between populations; for example, the mean urinary concentration of 2,5 DCP among the present sample was 206.5 vs. 6.05 ng/mL among the NHANES sample. Moreover, our study employed a prospective study design, while NHANES used a cross-sectional design. Further, findings revealed that triclosan was significantly associated with a lower odds of abdominal obesity 9 years later, in line with previous results demonstrating a similar inverse relationship between triclosan and waist circumference among women (42). Surprisingly, we did not find a significant association between BPA and MetS, in contrast to findings from cross-sectional studies conducted among Lebanese (13) and Korean women (14). However, our results are similar to those among a sample of Serbian women of reproductive age (43). Differences between studies may be due to various reasons, such as varying study designs (most prior studies were cross-sectional). Another reason could be differences in exposure levels between populations, with mean BPA levels being higher among other populations compared to the mean BPA level among the present sample. Furthermore, it is well-known that ethnicity is strongly correlated with MetS. However, it is less known if ethnicity modifies the relationship between EDCs and MetS. The differences in the results of our study among Mexican women, in comparison to other populations (13, 22), may be an indication that ethnicity is a modifier of the EDC-MetS association, and/or, that EDCs contribute to ethnic disparities in MetS. Another potential explanation for differences in findings could be related to the present study focusing on three chemical classes of EDCs, whereas other studies tend to focus on only one class of EDCs. Studies have demonstrated that under combined exposure to DBP and PBA, study outcomes of interest were drastically altered, which was suggestive of an additive or a synergistic effect (44). Given the focus of multiple classes of EDCs, it can be speculated that EDCs in the present mixture may have produced synergistic effects on MetS and its components. Thus, we cannot rule out possible synergistic effects of chemical mixtures. However, given our sample size, a mixture analysis was not ideal.

Previous research has proposed plausible mechanisms to explain positive associations between toxicants and MetS risk, including mechanisms whereby environmental exposures can disturb mitochondrial function (45). It has been well-documented that glucose and lipid metabolism pathways are dependent on mitochondria for generating energy. When mitochondrial dysfunction is present, ATP production and oxygen consumption are impaired, and reactive oxidative species (ROS) production is elevated, resulting in insulin resistance (IR). Likewise, one common mechanism widely used to explain the endocrine effect of triclosan and other EDCs on body weight relates to its interference with thyroid function *in vitro* and animals (46–49). Studies have revealed decreased synthesis and increased catabolism of thyroid hormone, resulting in lower circulating thyroid hormone levels and increasing weight (46–49), contrary to our findings. However, human data on endocrine disruption are lacking (50, 51). Another potential mechanism that must be considered when examining the present associations is the role of estrogen in regulating body weight and metabolic health (22). Human studies have demonstrated the protective role of estrogen against metabolic syndrome through various mechanisms, such as appetite suppression, the conversion of white fat to brown fat and estrogens preference for subcutaneous over visceral fat deposition to prevent expansion of visceral adipose tissue (22). Midlife is a period marked by a decrease in estrogen (5), which places women at an increased risk of metabolic disruption. Further, EDCs are known to interact with estrogen (10), thus we cannot rule out the role of estrogen when examining these associations.

Within this study among midlife women, we found inverse associations between individual toxicants and MetS syndrome components, not previously established in the literature. For example, contrary to existing research (11, 39, 52), we found ΣDEHP to be inversely associated with hyperglycemia, while triclosan was associated with lower odds of abdominal obesity. It is unclear why these inverse associations did not align with previous research; albeit, it is plausible that mechanisms outside of those previously mentioned drive associations. One potential mechanism that may explain our mixed findings is the ability for different EDCs to activate or inhibit PPARs (peroxisome proliferator-activated receptors) nuclear receptors, affecting metabolic homeostasis (53–55) and responsible for adipogenesis. It is worth mentioning that we have noted inverse associations between EDCs and obesity among the ELEMENT study participants during pregnancy, such that urinary ΣDEHP was inversely associated with weight at delivery (56).

Our study included several strengths. First, it employed a prospective study design to assess the long-term impact of 3 different toxicant classes and MetS. In contrast, other studies have mostly used cross-sectional study designs, whereby the exposure and outcome are measured at the same time. Second, to our knowledge, the present preliminary study is the first study conducted among a cohort of mid-life Latin American women. Moreover, the sample was likely generalizable to the Mexican population given that 34.3% of women enrolled in the present study were classified as having MetS, similar to a 2006 nationally representative sample of Mexico City adults (35.8%) (57). Limitations included using a small sample of women in midlife, which results in a reduction of statistical power. Secondly, the present study sought to determine associations between toxicants and MetS among women in midlife, which encompasses the menopausal transition. However, we did not objectively confirm menopausal status. Future research will collect steroid hormones to confirm menopausal status and will include collection of repeated assessments of exposure to provide more information on potential sources of exposure. Third, the toxicant levels were assessed using one spot urine sample that was collected in 2008. Moreover, the toxicants assessed in the present study have a short biological half-life (58), therefore toxicant levels may not necessarily represent chronic exposure. Thus, unless exposure remains consistent across time, it may not be appropriate to assume that exposure measured years before the outcome signifies chronic exposure. In alignment, due to funding restrictions we were unable to conduct exposure assessment 9 years later to monitor exposure over-time. Fourth, as with any observational study of this nature it impossible to establish the mechanisms that underlie the inferred associations and, therefore, determine causality. In conjunction, as with any epidemiologic study, there may be some unmeasured confounders which may bias effect estimates. Finally, due to potential differences in toxicant exposure frequency, duration, and intensity, various analytes were not detected among the present sample, while others were detected in high amounts. Therefore, our findings may not be generalizable to all women between the ages of 40–50 years.

The impact of EDCs on metabolic health risk among the general population is becoming more evident; however, few studies have implemented a longitudinal framework to determine the effects of EDCs on women in midlife. The present study found evidence indicating that phthalates (ΣDBP, MBzP, MCOP, and MEP), phenols (2,5 DCP and 2,4 DCP), and parabens (propyl-paraben) measured in 2008 were predictive of individual MetS components, in particular, hypertriglyceridemia and hypertension 9 years later. In contrast, a few phthalate metabolites and phenol analytes were inversely associated with MetS components (ΣDEHP and Triclosan). A future investigation conducted among the ELEMENT study should confirm our findings by including a larger sample size and determining the underlying mechanisms driving our findings. If replicated in prospective studies and supported by mechanistic research, our results suggest that lower exposure to certain phthalates, phenols, and parabens could lower several markers of metabolic risk among midlife women.

## Data Availability Statement

The datasets generated for this study are available on request to the senior author (karenep@umich.edu).

## Ethics Statement

The studies involving human participants were reviewed and approved by The Mexico National Institute of Public Health (INSP) and the University of Michigan Internal Review Boards approved all research protocols and procedures, and all participants provided informed consent. The patients/participants provided their written informed consent to participate in this study.

## Author Contributions

KP, MT-R, MT-O, BS, JT-O, EJ, and JG contributed to the study's conception and design. AM-G was responsible for oversight and implementation of fieldwork protocols. AZ performed the statistical analysis and wrote the first draft of the manuscript. EJ, MT-O, AB, JG, and KP provided oversight in the analysis and writing processes. KP and JT-O were responsible for funding acquisition. All authors contributed to the manuscript and approved the submission.

## Conflict of Interest

The authors declare that the research was conducted in the absence of any commercial or financial relationships that could be construed as a potential conflict of interest.
